# Surface colonization and subsequent development of infections with multi drug resistant organisms in a neonatal intensive care unit

**DOI:** 10.1186/s12941-019-0312-2

**Published:** 2019-03-20

**Authors:** Mary Dias, Juveyriya Saleem

**Affiliations:** Department of Microbiology, St. John’s Medical College, Sarjapur Road, Bangalore, Karnataka 560034 India

**Keywords:** Colonization, Neonatal intensive care unit, Methicillin resistant *Staphylococcus aureus*, Vancomycin resistant *Enterococcus* species, Multi drug resistant organisms

## Abstract

**Background:**

This study analyzes colonization of the neonates in a NICU and incidence of these colonized infants developing infections due to the colonizers.

**Methods:**

Over a 12 month period, samples (surface swabs and rectal swabs) were obtained from all the infants admitted to NICU. The samples were cultured and examined for the presence of colonizers and especially for multi-drug resistant organisms.

**Results:**

From the total 533 patients, 473 (89%) neonates acquired colonizers and 60 (11%) did not. Of the 473 (89%) colonized infants, 57 (12%) developed infections of whom 33 (58%) were infected from the same organism as the colonizer and 24 (42%) neonates developed an infection that was different from the colonizer. 416 (88%) infants did not develop any infection inspite of being colonized.

**Conclusions:**

The total numbers of babies contracting infection were more in the colonized group than the non-colonized. Other factors like gestational age and preterm may have played a role in development of infection in addition to colonization in these babies. Screening for the presence of MDRO colonization may be of limited use in predicting infections in the colonized individual. However, knowledge of their presence results in implementation of strict infection control practices. This along with judicious uses of antimicrobials effectively reduces infections from colonized bacteria and more importantly prevent their spread.

## Background

The infants admitted to the NICU are prone to acquire nosocomial infections owing to their vulnerable defense mechanisms. In addition, other factors like gestational age, use of antimicrobials, invasive procedures, frequent contacts with healthcare workers and infectious agents colonizing skin and mucous membrane of the infants also increases their chance of acquiring infections [1, 2]. The problem is further aggravated if these colonizing organisms are drug resistant with studies showing that neonates colonized with MRSA, VRE and ESBL producing *Escherichia coli* and *Klebsiella* have a higher chance of developing infections compared to those who are not colonized [3–7].

The neonatal intensive care unit (NICU) of St. John’s Medical College and Hospital (SJMCH) is a referral center for neonates from all part of Bangalore and neighboring states like Andhra Pradesh and Tamil Nadu. Routine surveillance by surface swab screening is done to rule out colonization with MDROs to institute isolation precautions to prevent spread and therefore potential infections between colonized and the noncolonized babies. We undertook this study to see if there was a relationship between colonization of the infants with multi-drug resistant organisms and subsequent development of infection in them.

## Methods

**Fig. 1:**
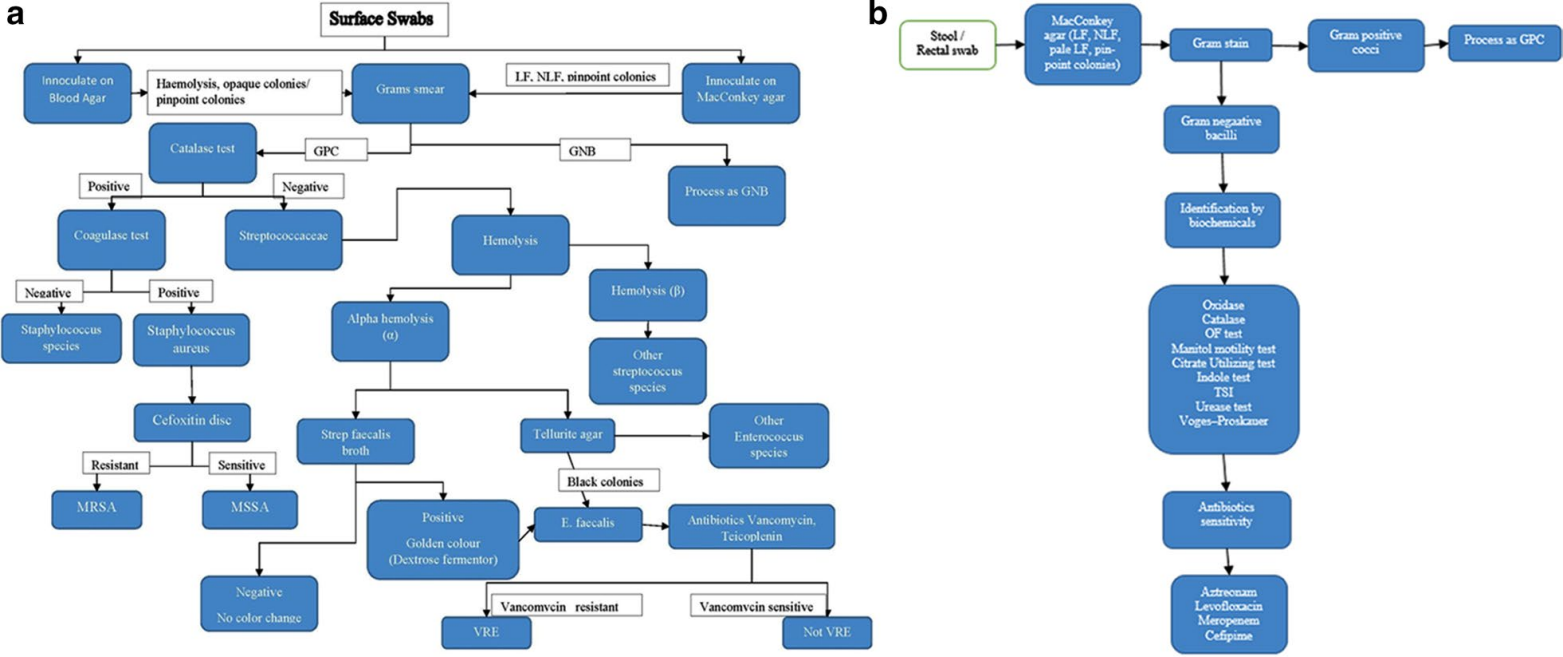
Flowchart of processing surface swabs and rectal swabs. *GPC* Gram positive cocci, *GNB* gram negative bacilli, *MDR GNB* multi drug resistant gram negative bacilli, *LF* lactose fermenters, *NLF* non lactose fermenters

Surveillance samples (Surface swabs, stool samples/rectal swabs) were obtained from neonates admitted to the NICU at St. John’s Medical College Hospital from 1st January 2017 to 31st December 2017, upon admission and once a week if the length of stay was more than 7 days. These samples were cultured to look for the presence of colonizers. Identification of the organisms, antibiotic susceptibility testing and multi-drug resistant organisms were determined as per the flow charts (Fig. 1). At the same time, these babies were followed up to look for the development of culture positive infections (blood stream infections, respiratory infections, urinary tract infections, endocarditis, meningitis etc.) from the Laboratory information System and review of the patient’s medical records.

The samples taken from stool/rectal swabs were inoculated on MacConkey agar and 5% sheep blood agar, and the surface swabs were inoculated on 5% sheep blood agar. The inoculated media were kept overnight at 37 °C for incubation and observed for growth. Gram smears were made from the colonies and biochemical tests were used to further identify these organisms. Antibiotic susceptibility testing was done Kirby-Bauer Disc diffusion method. The antibiotics used were vancomycin, teicoplanin for *Enterococcus*, Meropenem, Levofloxacin, Aztreonam, Cefipime for Gram negative bacilli (GNB) and Cefoxitin for *Staphylococcus*.

The GNB that showed resistance to Meropenem alone or Levofloxacin, Aztreonam, Cefipime in combination with were considered as multi-drug resistant Gram negative bacilli (MDR GNB). *Staphylococcus aureus* that showed cefoxitin resistance were considered as methicillin resistant *S. aureus* (MRSA). *Enterococcus* that showed vancomycin resistance was taken as vancomycin resistant *Enterococcus* species (VRE).

## Results

In the present study, samples from neonates in NICU were screened for colonizers and subsequently followed up to see whether these colonizers developed into infections in the neonates. A total of 938 (from 533 patients) rectal samples and 884 (from 492 patients) surface swabs were taken during this study period.

Colonizers were found on 473/533 (89%), and those who were not colonized were 60/533 (11%). Duplicate isolates from the same neonate were excluded. Majority of the colonizers found in the study were Coagulase negative *Staphylococcus*, *E. coli* and *Klebsiella* species with a frequency of 366 (37.5%), 249 (25.5%) and 210 (21.5%), respectively. A total of 68 (7%) of the total isolates were found to be drug resistant. Of these 36 (8%) were from rectal swabs and 32 (6%) cases from surface swabs. The isolates that showed resistance were *Klebsiella* species, *E. coli* and Methicillin resistant *Staphylococcus* species with a frequency of 28 (2.9%), 18 (1.8%) and 13 (1.3%), respectively (Table 1).

**Table 1:** Rectal and surface swabs samples against bacterial species

Colonizers	Rectal swabs					Surface swab					Grand total					
	MDR	S	Total	MDR%	S%	MDR	S	Total	MDR%	S%	MDR total	MDR%	S Total	S%	G total	%
Gram negative baccilli																
*Citrobacter freundii*	0	1	1	0%	100%		1	1	0%	100%	0	0.0%	2	0.2%	2	0.2%
*Citrobacter* spp	0	1	1	0%	100%			0			0	0.0%	2	0.2%	2	0.2%
*E. coli*	14	179	193	7%	93%	4	52	56	7%	93%	18	1.8%	231	23.7%	249	25.5%
*Enterobacter* spp	0	4	4	0%	100%		1	1	0%	100%	0	0.0%	5	0.5%	5	0.5%
*Proteus* species	0	2	2	0%	100%			0			0	0.0%	2	0.2%	2	0.2%
*Klebsiella* spp	18	127	145	12%	88%	10	55	65	15%	85%	28	2.9%	182	18.7%	210	21.5%
MDR	1		1	100%	0%			0			1	0.1%	0	0.0%	1	0.1%
NF GNB	3	3	6	50%	50%	3	1	4	75%	25%	6	0.6%	4	0.4%	10	1.0%
*P. mirabilis*	0	3	3	0%	100%		4	4	0%	100%	0	0.0%	7	0.7%	7	0.7%
*P. vulgaris*	0	1	1	0%	100%		1	1	0%	100%	0	0.0%	2	0.2%	2	0.2%
*Proteus retgeri*	0	2	2	0%	100%		2	2	0%	100%	0	0.0%	4	0.4%	4	0.4%
*Providencia rettgeri*	0	1	1	0%	100%			0			0	0.0%	1	0.1%	1	0.1%
*Pseudomonas aeroginosa*	0	2	2	0%	100%		1	1	0%	100%	0	0.0%	3	0.3%	3	0.3%
*Serratia marcescens*			0				1	1	0%	100%	0	0.0%	1	0.1%	1	0.1%
Coaulase negative *Staphylococcus* species	0	36	36	0%	100%	0	330	330	0%	100%	0	0.0%	366	37.5%	366	37.5%
*Enterococcus faecalis*	0	33	33	0%	100%	0	26	26	0%	100%	0	0.0%	59	6.1%	59	6.1%
*Enterococcus* species	0	10	10	0%	100%	0	10	10	0%	100%	0	0.0%	20	2.1%	20	2.1%
Methicillin resistant *Staphylococcus aureus*						13		13	100%	0%	13	1.3%	0	0.0%	13	1.3%
Methicillin sensitive *Staphylococcus aureus*							2	2	0%	100%	0	0.0%	2	0.2%	2	0.2%
*Staphylococcus aureus*							14	14	0%	100%	0	0.0%	14	1.4%	14	1.4%
Vancomycin resistant *Enterococcus species*						2		2	100%	0%	2	0.2%	0	0.0%	2	0.2%
Grand total	36	406	442	8%	92%	32	501	533	6%	94%	68	7%	907	93%	975	99.8%

Among the 473 babies who were colonized, 57 (12%) developed infections. Interestingly it was seen that in 24 of 57 (42%) colonized neonates the organisms causing infections were not same as the colonizers residing on them. (Table 2).

**Table 2:** Distribution of colonizers

Category	N	%
Total number of patients screened	533	100%
Patient with colonizers	473	89%
With infections	57	12%
With same colonizer developing Infection	33	58%
With colonizer developing different Infection	24	42%
Without infection	416	88%
Patient without colonizers	60	11%
With infections	8	13%
Without infection	52	87%

Among the babies who had infection with the same organism as the colonizer, 6/33 were multi drug resistant and the rest 27/33 were sensitive to majority of the antibiotics. The sensitive organisms were Coagulase negative *Staphylococcus* species, *Enterococcus faecalis*, *Klebsiella* species, *E. coli*, *S. aureus,* NFGNB and *Pseudomonas aeruginosa* (Table 3).

**Table 3:** Total colonizer with the same infection (organism wise) and antibiotic sensitivity

Organism	Frequency	%	Sensitivity (%)
Coagulase negative *Staphylococcus* species	20	52.6%	60.6%
*Enterococcus faecalis*	3	7.9%	9.1%
*Klebsiella* species	7	18.4%	9.1%
*Escherichia Coli*	5	13.2%	12.1%
*Staphylococcus aureus*	1	2.6%	3.0%
Non fermenting gram negative bacilli	1	2.6%	3.0%
*Pseudomonas aeruginosa*	1	2.6%	3.0%
Total	38	100.0%	100.0%

Among the 473 babies who had acquired colonization, the multi-drug resistant organisms isolated were MRSA, VRE and MDR GNB with a frequency of 13, 2 and 46 respectively. However none of the MRSA or VRE developed into an infection in the neonates who were colonized with them although it was seen that 5 (11%) patients developed infection with MDR GNB. *Klebsiella* species and *E. coli* was isolated in 4 and 1 infants respectively.

65 neonates developed an infection in the study period irrespective of their colonization status. These neonates with infections were divided into three groups for analysis. Group A: Infective agent same as colonizer N = 33, Group B: Infective agent different from colonizer N = 24 and Group C: No colonizers but with infection N = 8. On comparing these three groups, it was seen that there were higher percentage of preterm babies in Group A (19 of 33, 59%) and Group B (12/24, 48%) that is babies who had colonization and infection than Group C (2/8, 25%).

The birth weight of the infants was lower in Group A when compared to the other two groups. Administration of antimicrobials before a positive culture was also higher in Group A than the other two groups.

However it was observed that major causes of admission into the NICU like respiratory distress syndrome, neonatal jaundice, asphyxia, and sepsis features were almost similar in distribution among the 3 groups. Also there was no significant difference in the type of delivery between the three groups (Table 4).

**Table 4:** Distribution of neonates who developed infections and their clinical details

Category	Group A: same colonizer developing same infection (n = 33)	%	Group B: same colonizer developing different infection (n = 24)	Group C: no colonizer but with infection (n = 08)	%
Term					
Pre-term	19	59%	12	2	25%
Term	13	41%	13	6	75%
Birth weight					
Low	21	70%	14	4	44%
Normal	9	30%	10	5	56%
Type of delivery					
C-section	19	61%	15	4	57%
Normal	12	39%	7	3	43%
Antibiotics administered before positive culture					
Yes	20	61%	5	1	13%
No	13	39%	19	7	88%
Cause of admission					
Respiratory distress syndrome	18	55%	9	1	13%
Asphyxia	7	21%	5	1	13%
Seizure	4	12%	4	2	25%
Sepsis features	14	42%	3	1	13%
Feeding intolerance	3	9%	1	0	0%
Apnoea	3	9%	2	1	13%
Neonatal jaundice	7	21%	6	3	38%
Pneumoniae	5	15%	2	1	13%

Statistical analysis were done using the Chi square test and student T test for the occurrence of colonization and development of infection among the colonizing and non-colonizing group of infants and the *p* value obtained was 0.079 and not statistically significant, although the total number of babies contracting infection were more in the colonized group than the non-colonized suggesting that other factors like gestational age and preterm may have played a role in development of infection other than bacterial colonization in these babies.

## Discussion

Colonization is a natural process that occurs in an infant right after birth [8–14]. At times these colonizers move to become causative agents of infections. This is especially the case when the defense mechanism of the host is impaired due to prematurity. We undertook the present study to look for the colonization versus infections in an Intensive care area of a tertiary care hospital.

Samples were initially screened for colonizers among all the babies admitted. Subsequently all these babies were followed up longitudinally till they left the NICU to look for the development of infections in them. A total of 938 (from 533 patients) rectal samples and 884 (from 492 patients) surface swabs were taken.

The colonized infants were 473 (89%), and those who were not colonized were 60 (11%). Majority of the colonizers found in the study were Coagulase negative *Staphylococcus*, *E. coli* and *Klebsiella* species with a frequency of 366 (37.5%), 249 (25.5%) and 210 (21.5%), respectively. A study by Rotimi et al. [15] on the development of bacterial flora of premature neonates, showed that majority of the colonizers present in the rectal swabs contained *E. coli*.

88.7% of the babies screened in the study period were found to be colonized, and of these 12% developed infections from the same organisms that were isolated as colonizers. Though the colonization rates were similar as in a study by Leonard et al. [16] who showed that 90% of the neonates were colonized, they had a higher infection rate in the colonized neonates (35%) than the present study.

Among the 57 colonized infants, 33 (58%) developed infections that were the same as the colonizers and 24 (42%) developed infections that were not same as the colonizers in the babies. In addition, the babies who had infection with the same organism as the colonizer, six isolates were multidrug resistant and the rest were sensitive to majority of the antibiotics.

In the study, we found that several multi-drug resistant organisms reside as colonizers in the neonates. Among the 473 patients with colonization, the multi-drug resistant organisms isolated were MRSA, VRE and MDR GNB with a frequency of 13, 2 and 46, respectively. However, none of the MRSA or VRE developed into an infection in the neonates who were colonized. But, it was seen that five (11%) patients developed infection for MDR GNB. Previous study groups have stated that they have ended standard culturing and nursing in isolation of NICU patients based on the observations they found in the study that showed a low prevalence of highly resistant microorganism (HRMO) and low incidence of bacterial infections in neonates after transfer from a NICU [17–24]. We also saw that not all infections that developed in the neonates were the same as the colonizers residing on them and this was seen in 42% colonized infants. Author Jolley et al. [11] state that the surface cultures in predicting the organisms responsible for infection are ineffective and not economical as most NICU’s have had an antibiotic policy which covers the common microbes and the treatment is not often changed as a result of pathogen isolated from surface culture.

There were a total 65 neonates who developed an infection during the study period irrespective of their colonization status. These neonates with infections were divided into three groups, Group A: the Infective agent same as colonizer (N = 33), Group B: Infective agent different from colonizer (N = 24) and Group C: No colonizers but with infection (N = 8).

On comparing these three groups, it was seen that higher percentage of preterm babies, lower birth weight and exposure to antibiotics Group A (19/33, 59%) and Group B (12/24, 48%) who had colonization and infection than Group C (2/8, 25%). This finding was similar to previous studies in NICUs that revealed increased early colonization in preterm newborns and other risk factors like low birth weight and previous antibiotic administration [3, 8].

Lastly the occurrence of colonization and development of infection though higher in the colonized than in the non-colonized group of infants, was not found to be statistically significant (p value = 0.079) suggesting other factors may also have played a role on the development of infections in these babies.

## Conclusions

The findings in our study along with the existing evidences emphasize the gastrointestinal tract and other body surfaces to be essentially sterile at birth and is subsequently colonized by microbes acquired from the mother and the surrounding environment. The neonates that are required to stay in the hospital for longer periods, especially in the NICU are already at a higher risk to develop an infection as their host defense mechanism are not well developed at this stage and some commensals/colonizers may become opportunistic pathogens. Repeated and/or prolonged courses of antibiotic exposure have resulted in an increase in the prevalence of antibiotic-resistant organisms such as methicillin-resistant *S. aureus*, vancomycin-resistant *Enterococcus*, and multidrug-resistant Gram-negative rods. These MDROs now prevalent in the environment of the hospital and become the colonizer/commensal on the babies.

Screening of the presence of MDRO colonization may be of limited use in predicting infections in the colonized individual. However knowledge of their presence results in implementation of strict infection control practices and effective hand washing. These along with judicious use of antimicrobials are the most important methods to effectively reduce infections from colonized bacteria and more importantly prevent further spreading.
